# Supplementary material for: Population genomics of hypervirulent Klebsiella pneumoniae clonal-group 23 reveals early emergence and rapid global dissemination
Source: Nat Commun. 2018 Jul 13;9:2703. doi: 10.1038/s41467-018-05114-7 (PMC6045662; doi:10.1038/s41467-018-05114-7)
Supplement: Supplementary file 1 — Supplementary Information [file 41467_2018_5114_MOESM1_ESM.pdf]

SUPPLEMENTARY INFORMATION

Population genomics of hypervirulent *Klebsiella pneumoniae* clonal-group 23 reveals early emergence and rapid global dissemination

Lam and Wyres *et al.*

<u>**Index**</u>

1. Supplementary Data – see separate Excel files
Supplementary Data 1: Isolate information
Supplementary Data 2: Nucleotide mutations defining the globally distributed CG23-I sublineage
Supplementary Data 3: Nucleotide mutations defining the horse-associated sublineage

2. Supplementary Figures – p2

3. Supplementary Methods – p10

4. Supplementary References –p13

Supplementary Figures

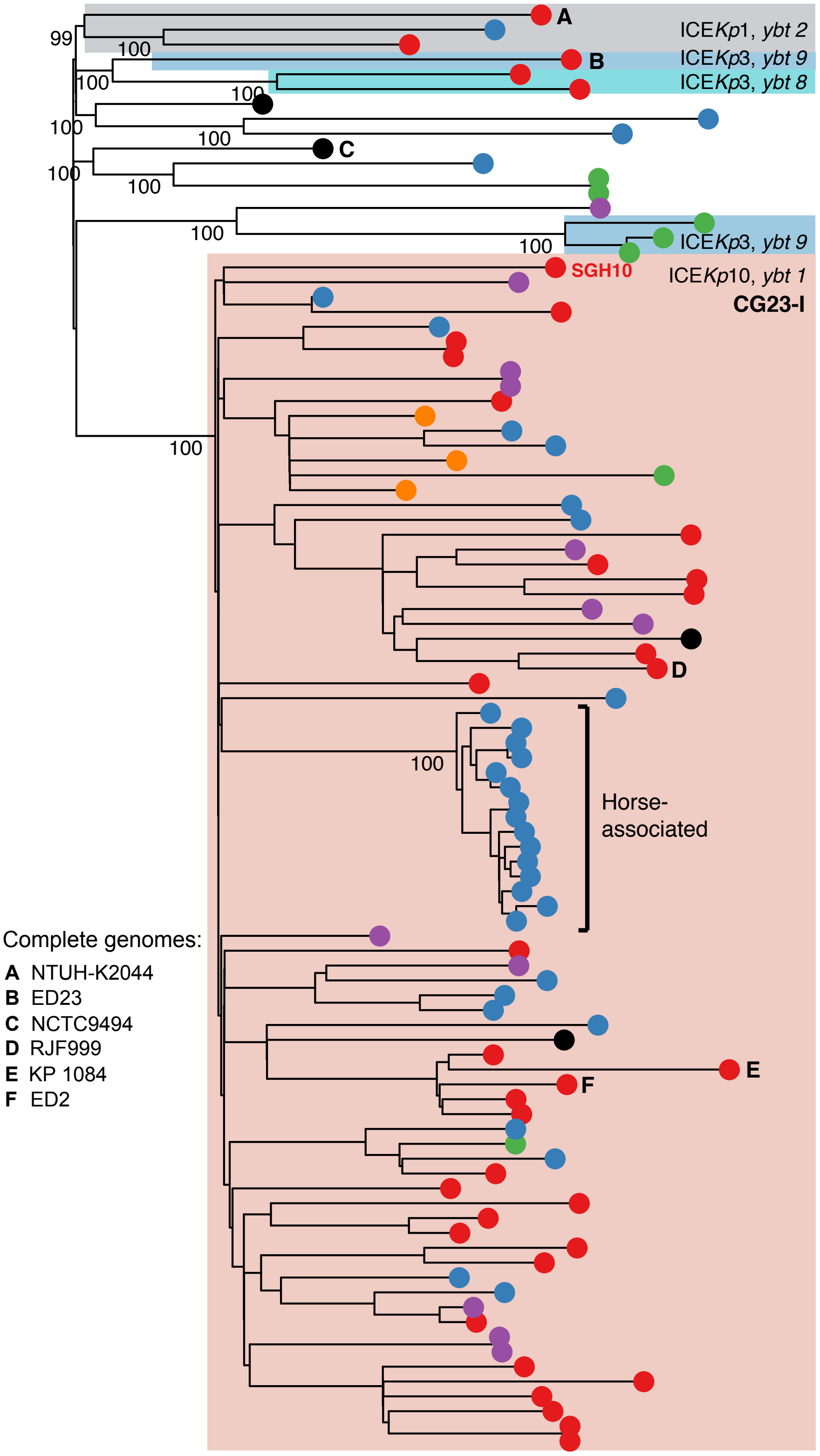

Supplementary Figure 1: Maximum likelihood phylogeny for CG23
The relationships between 97 CG23 isolates as determined by maximum likelihood (best of 5 runs each 100 bootstraps). Bootstrap support values >80 are shown for nodes discussed in the main text and/or associated with the acquisition of ICE*Kp*s. Tips are coloured by region of isolation as defined in **Fig. 1**, and the tips corresponding to SGH10 and the other completely

sequenced chromosomes (A-F; see legend for assignment) labelled accordingly. Shading indicates the presence of ICE*Kp* and labelled with the corresponding ICE*Kp* variant and *ybt* lineage.

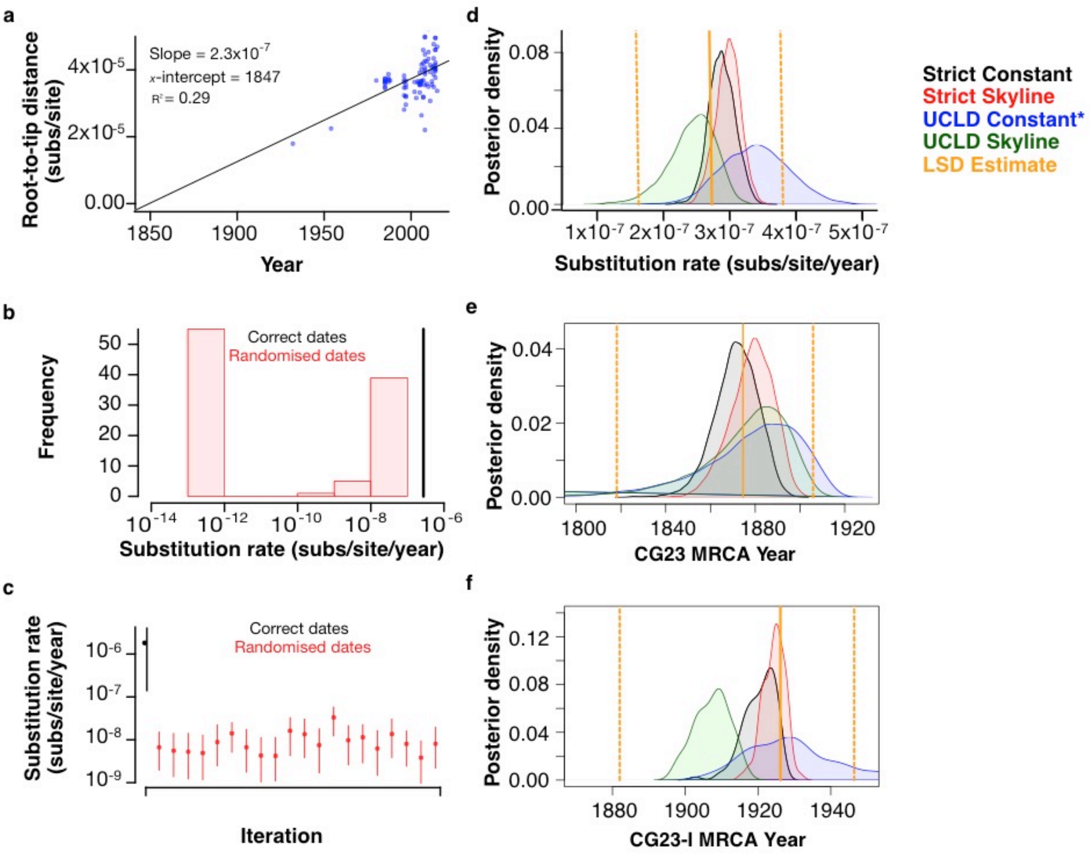

Supplementary Figure 2. Evidence for temporal signal with agreement of dating methods and models

a) Correlation between root-to-tip distance (ML phylogeny) and year of isolate collection. **b)** Substitution rate estimates generated by the LSD least squares method with the correct isolate collection dates (black) and randomised dates (red, n = 100 tests). **c)** Posterior distributions for substitution rate estimates generated by the Bayesian method implemented in BEAST with the correct isolate collection dates (black) and randomised dates (red, n = 20 tests). **d, e and f)** Distributions of estimates for substitution rates, time to most recent common ancestor (TMRCA) for all CG23 and TMRCA for CG23-I, respectively. Distributions for each of four different Bayesian models are distinguished by colour as indicated, with the model used for the phylogeny in **Fig. 1, Supplementary Fig. 6** and **9** marked with an asterisk. Estimates from the LSD method are shown in yellow +/- 95% confidence interval.

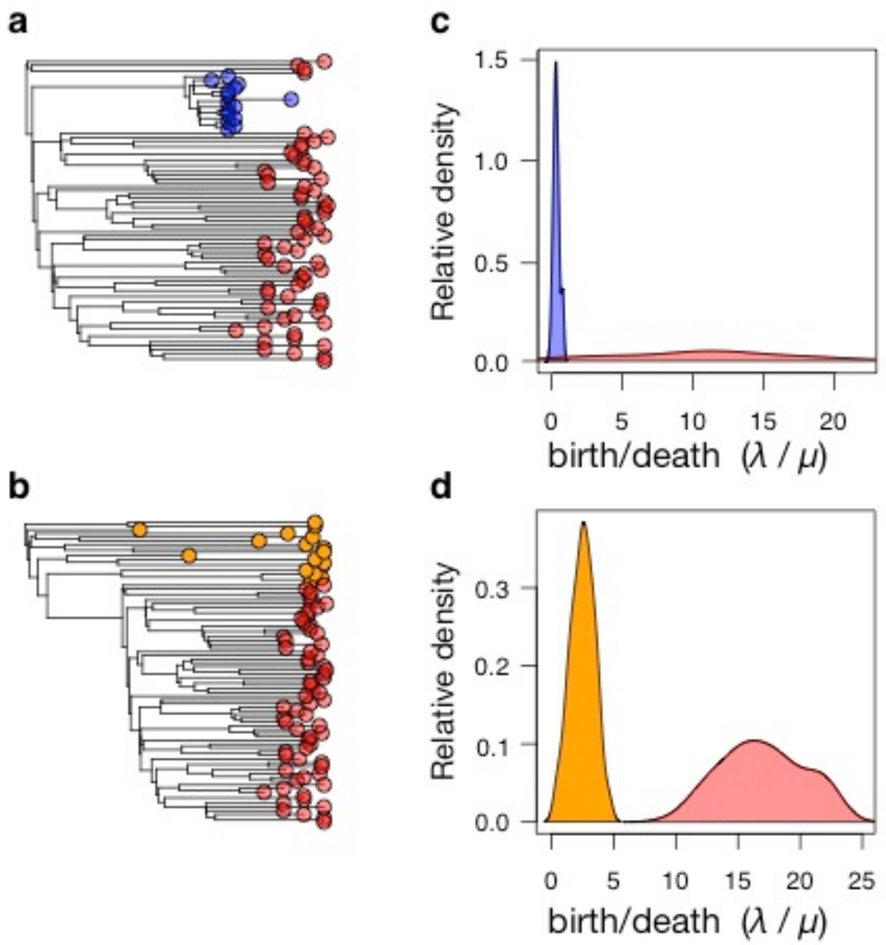

Supplementary Figure 3. Lineage diversification rate comparisons
Phylogenies are shown with tips coloured by sample stratification; **a)** human-associated CG23-I isolates (red) and horse-associated isolates (blue); **b)** human-associated CG23-I isolates (red) and non-CG23-I isolates (orange). Densities show the distributions of birth/death estimates from a sample of 100 trees from the posterior distribution of trees estimated in BEAST for; **c)** human-associated CG23-I isolates (red) and horse-associated isolates (blue); **d)** human-associated CG23-I isolates (red) and non-CG23-I isolates (orange).

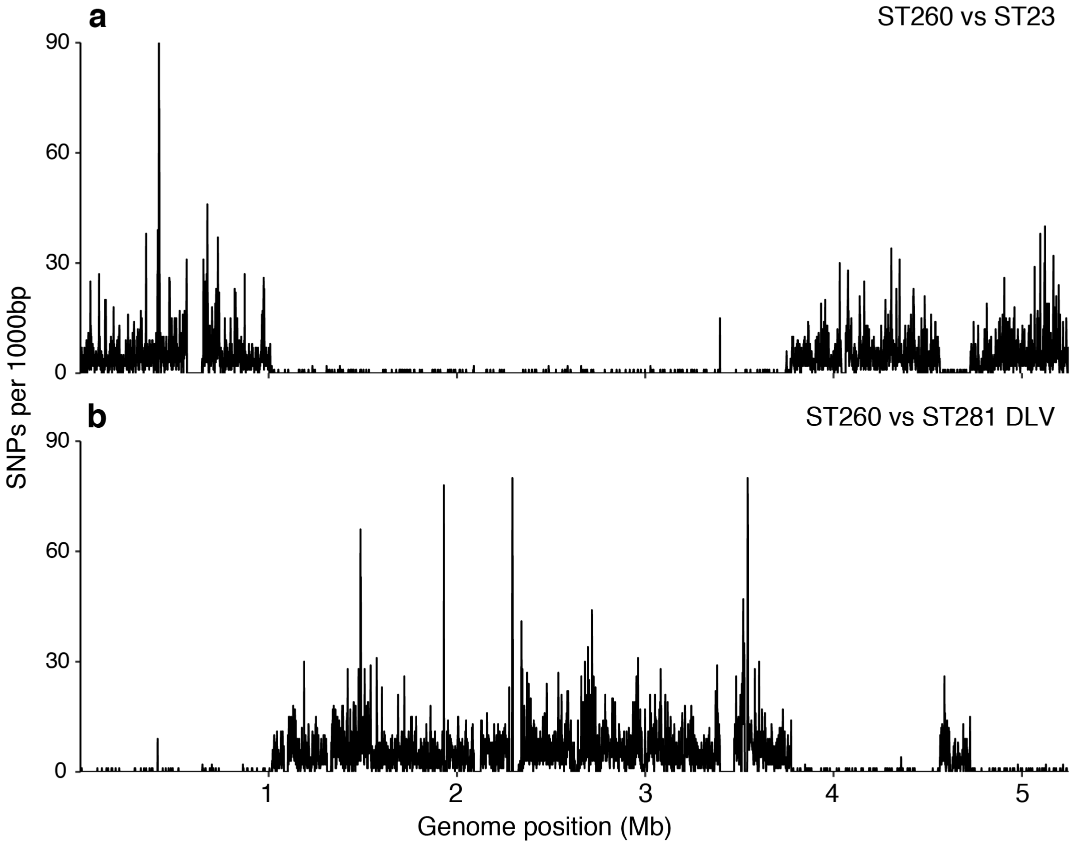

Supplementary Figure 4: Nucleotide polymorphisms distinguishing ST260 isolate CAS686 from (a) ST23 and (b) ST281-like double locus variant (DLV) isolate genomes. The number of pairwise single nucleotide polymorphisms (SNPs) per 1000bp is shown relative to position in the NTUH-K2044 (ST23) reference genome.

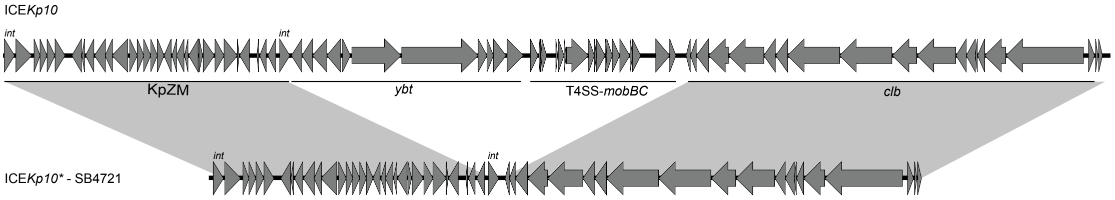

Supplementary Figure 5: Structure of ICE*Kp10 deletion variant in strain SB4721**
Upper tract shows the genetic structure of the *ybt+clb+* ICE*Kp10*, with the core Zn^{2+}/Mn^{2+} metabolism (KpZM), yersiniabactin synthesis (*ybt*), T4SS-*mobBC* mobilisation and colibactin synthesis (*clb*) modules indicated. Lower tract shows the structure of the ICE*Kp10** deletion variant. Grey shading indicates homologous sequence regions.

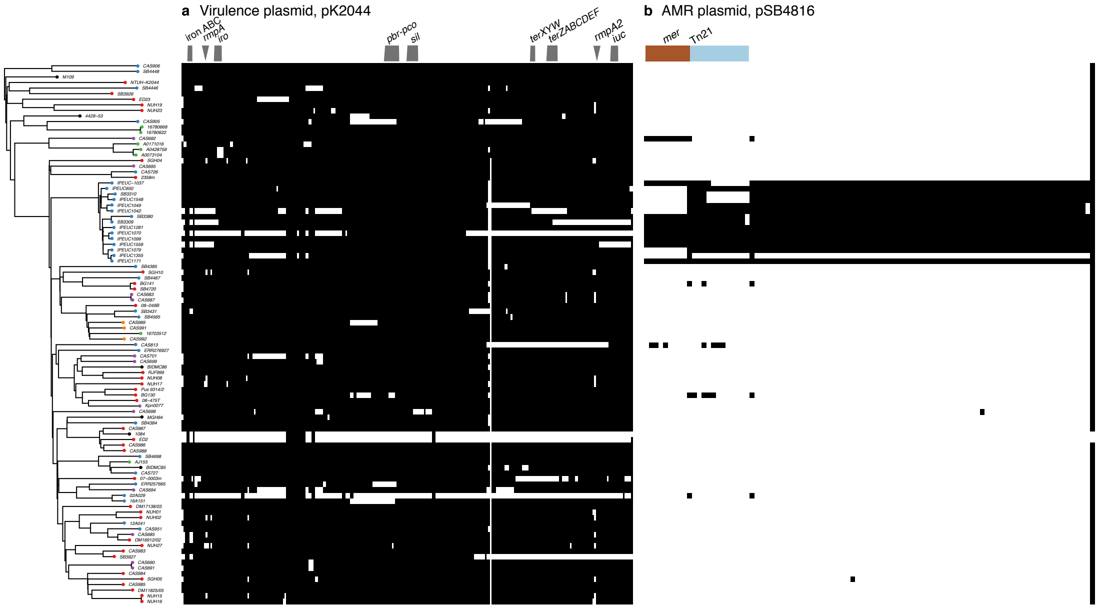

Supplementary Figure 6: Conservation of the pK2044 and pSB4816 plasmids
Chromosomal Bayesian phylogeny for CG23 (left) as shown in **Fig. 1**. Heatmaps indicate the conservation of genes annotated in **a)** the pK2044 virulence plasmid and **b)** pSB4816 AMR plasmid. Conservation was determined by read mapping to the reference (coverage ≥90%, depth ≥5). Regions of interest are indicted by coloured blocks: iron ABC; membrane-bound iron (III) transport system, *rmpA/rmpA2*; regulator of mucoid phenotype genes, *iro*; salmochelin operon, *pbr-pco*; copper resistance operon, *sil*; silver resistance operon, *ter*; tellurite resistance operon, *iuc*; aerobactin operon, *mer*; mercury resistance operon.

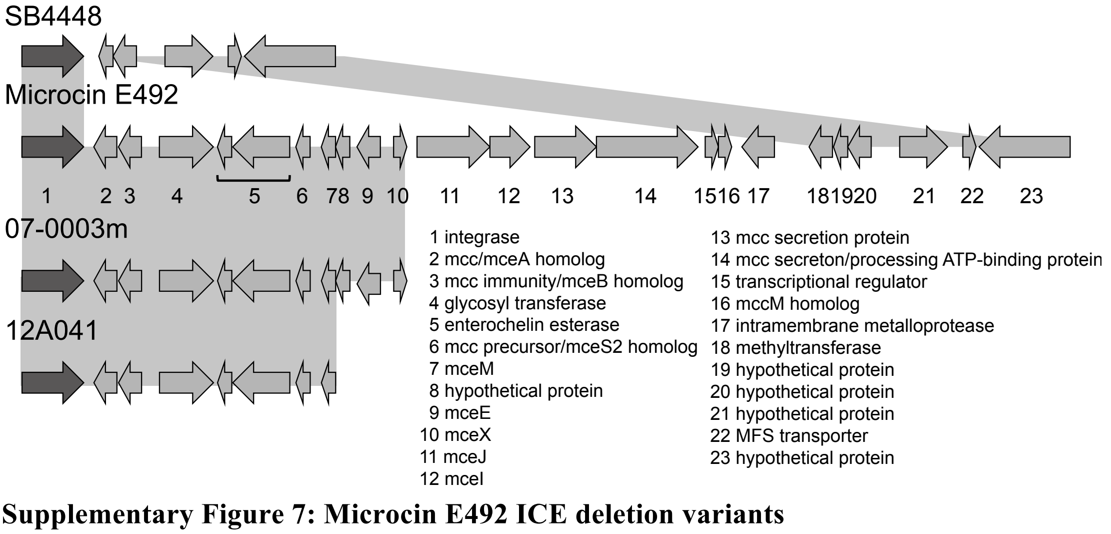

Supplementary Figure 7: Microcin E492 ICE deletion variants
Genetic structure of the microcin E492 ICE compared to the microcin ICE in each of the three partial deletion variants; 07-0003m, SB4448 and 12A041. Various portions of the right hand side were deleted from the ICE in strains 07-0003m and 12A041 contrasting to the middle region deleted in SB4448.

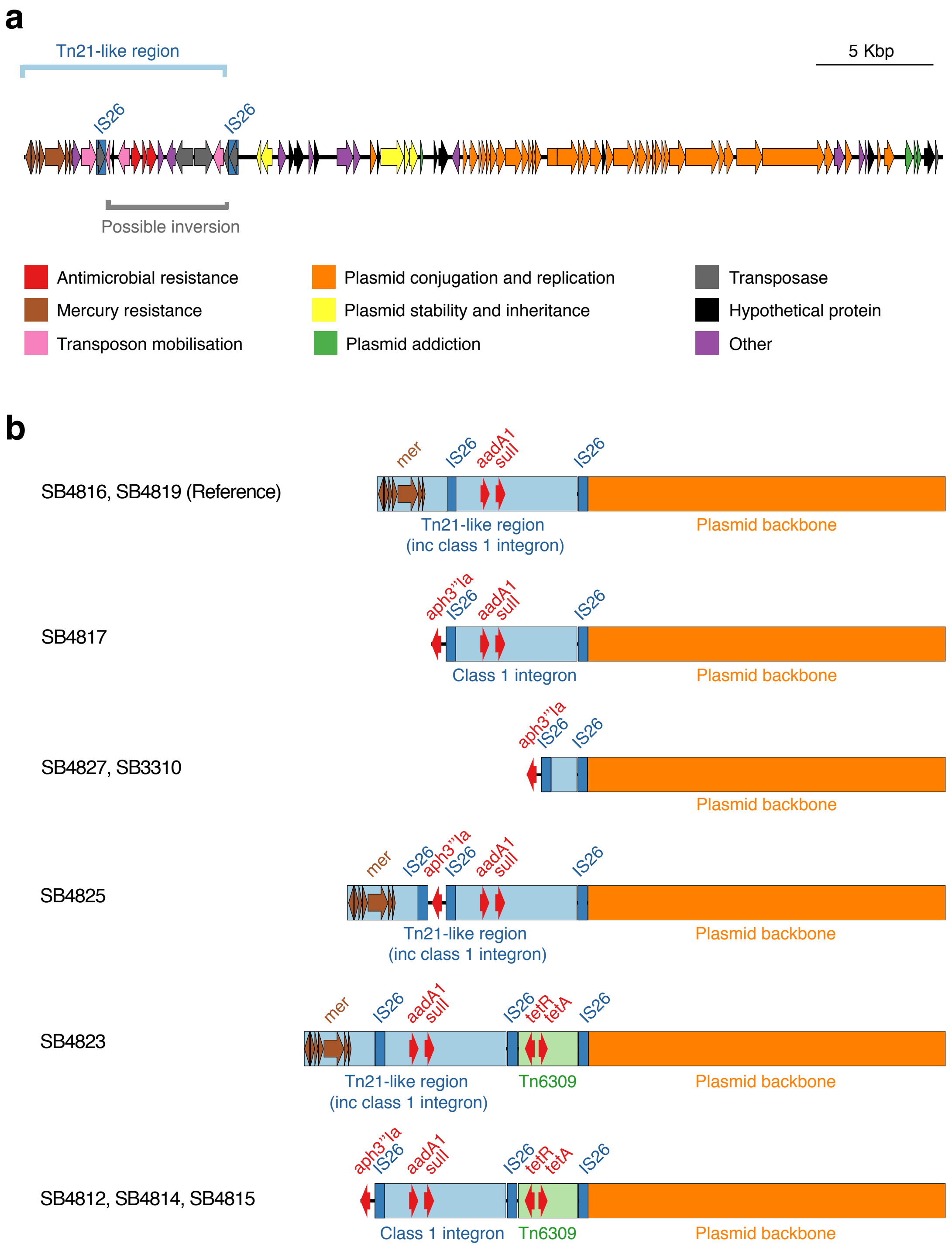

Supplementary Figure 8: Equine-associated AMR plasmid pSB4816
a) Novel plasmid pSB4816, with putative coding regions represented as arrows colour coded by functional grouping. The Tn21-like region is marked. **b)** pSB4816 variants. The Tn*21*-like region (light blue) containing a class 1 integron, mercury resistance operon (*mer*, brown), *aadA1* and *sulI* AMR genes (red) are variably present. Three variants contained the additional *aph3'1a* aminoglycoside resistance gene and two contained the Tn*6309* transposon (green) harbouring *tetR* and *tetA*. Variation in the plasmid backbone region is not shown.

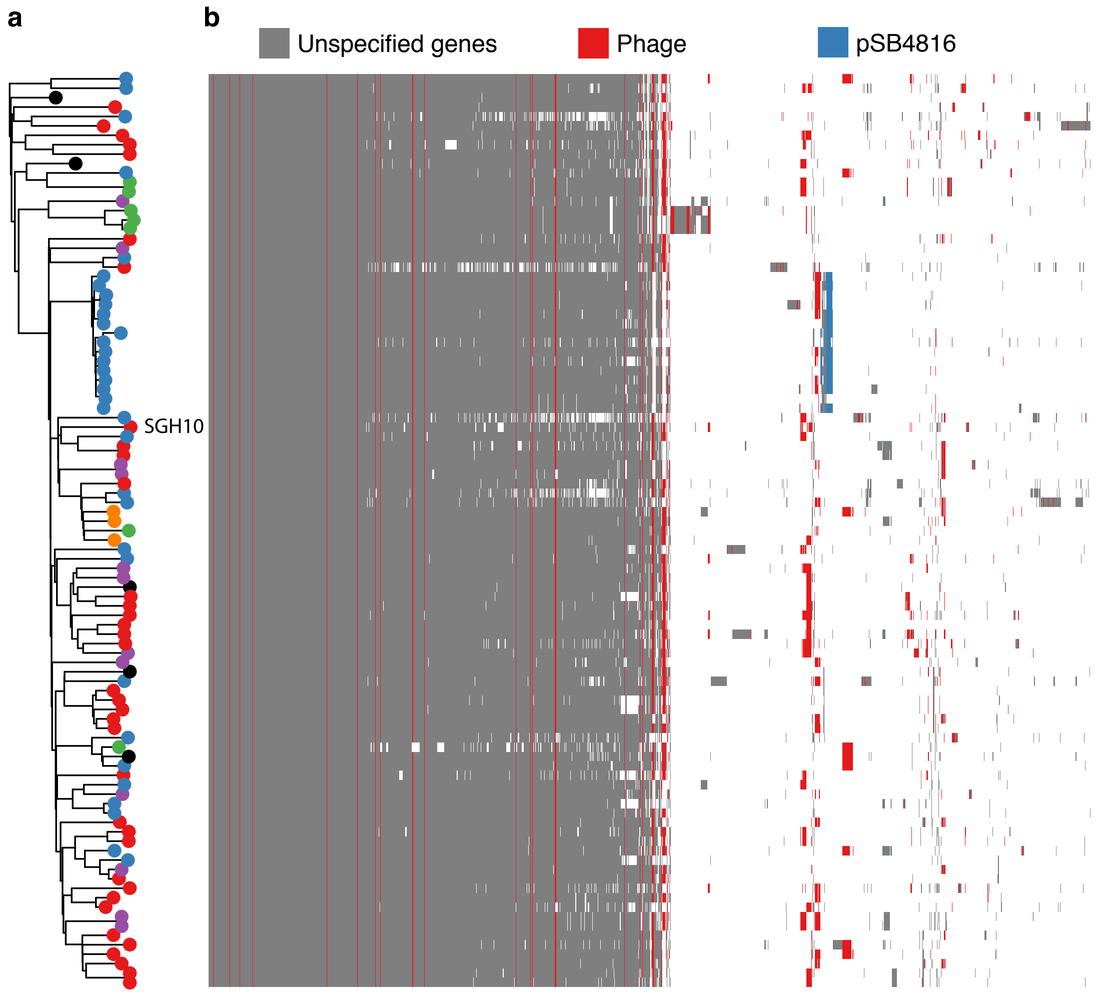

Supplementary Figure 9: CG23 core and accessory genome content
The pan-genome content of the CG23 isolates, comprising 9663 protein-encoding sequences, is plotted against (**a**) the Bayesian phylogeny (from **Fig. 1**), with the presence or absence of sequences in each CG23 genome represented by a coloured or white vertical band respectively shown in (**b**). Sequences that match complete phage sequences extracted from PHAST or plasmid pSB4816 are coloured red and blue, respectively.

Supplementary Methods

Virulence assessment of reference strain SGH10
Eight healthy 8-week-old female C57BL/6 mice were first treated with 2.5 mg ampicillin via oral gavage for 5 consecutive days. After 24 hours, the mice were infected with 10^8 colony forming units (CFU) of strain SGH10 in 100 μl PBS via oral gavage. The mice were euthanized 48 hours following infection and the bacterial burdens from the liver, lungs and spleen as well as that present in the stool were quantified.

Genome finishing for reference strain SGH10
Strain SGH10 was grown in LB broth overnight at 37°C prior to DNA extraction using Agencourt Genfind v2 (Beckman Coulter). Long reads were generated using a 1D Ligation library (SQK-LSK108) using Native Barcoding (EXP-NBD103) and sequenced on a MinION R9.4 flow cell (Oxford Nanopore Technologies) as previously described[1]. Base calling was performed using Albacore version 2.0.2 and yielded 2.4 Gbp of data (300,767 reads, mean length 7,984 bp, N50 length 12,707 bp). Additional short (150 bp) paired end reads were generated on the Illumina MiniSeq platform following library preparation with the Nextera DNA Flex kit, yielding 3,062,668 additional reads. We used Unicycler [2] to generate a hybrid assembly using all available short and long reads.

We used Unicycler v0.4.4-beta (20) to perform a hybrid assembly with our Illumina MiniSeq reads and the full set of Nanopore 1D reads. We then polished the assembly using Nanopolish v0.8.4 [3], using its methylation-aware setting. A second hybrid assembly was then performed with Unicycler, with the Nanopolish output provided as an existing long read assembly. This two-stage assembly was to ensure that for any regions of the genome not well covered by Illumina reads, the sequence was filled with best possible Nanopore read consensus. We then used Unicycler's polishing tool to reduce the number of sequence errors in repetitive regions of the genome.

To facilitate manual investigation, we aligned both available Illumina read sets: the previously available reads (accession SRR5082357) and our in-house MiniSeq reads (accession SRR6307304). FreeBayes [4] was used to search for positions in the genome where the two read sets disagreed. One key difference was found in the *wcaJ* gene of the K locus, where our MiniSeq reads contained a single base insertion (which caused a frameshift and presumed loss of function) but the first read set did not. We hypothesise this represents an adaptation to growing in culture – the K locus is costly and less valuable outside of a host environment. For our genome, we therefore changed this gene to the intact version of the first read set. Loss of virulence in culture may be a common phenomenon and demands further study.

Identification of nucleotide variants for phylogenetic inference
Sequence reads were mapped to the NTUH-K2044 reference chromosome (accession: AP006725) and single nucleotide variants were called using the RedDog v10b pipeline (https://github.com/katholt/reddog) which employs Bowtie 2 [5] and SAMTools v1.3.1[6]. SAMTools wgsim was used to simulate 100 bp paired end reads (without errors) for the 9 genomes that were available only as assemblies, to allow these genomes to be included in the same analysis with the Illumina read sets. Variant sites were filtered such that only those that met the following criteria were accepted: unambiguous consensus allele calls for which phred quality ≥30 and depth ≥5 reads but <2-fold mean read depth, and for which there was no evidence of strand bias. Base-calls with phred quality ≥20 were extracted from all genomes at all variant sites, and positions for which ≥95% genomes contained a phred ≥20 base-call were considered core loci and retained for phylogenetic anlaysis. Variant sites located within a putative phage region identified by PHAST [7] (positions 2089398 – 2118283 in NC_012731.1) or the *ybt*-containing ICE*Kp* (positions and 3395820 - 3472027) were excluded from phylogenetic analysis

Bayesian molecular clock analyses

We first used TempEst [8] to investigate the relationship between root-to-tip distances in the ML tree and year of isolation. We then used two different methods to infer the evolutionary rate and timescale. The first method consists of least-squares dating, implemented in LSD v0.3 [9], using as input the ML tree and year of isolation data. The hybrid CAS686 genome and five CG23 sequences with uncertain sampling times were excluded from this analysis.

The method implemented in LSD does not account for phylogenetic uncertainty and it assumes a strict molecular clock. To relax these assumptions we also analysed the data using the Bayesian framework in BEAST v1.8 [10], which implements a range of molecular-clock models, and allows co-estimation of the phylogenetic tree, evolutionary rates, and other parameters. For this analysis we included the five samples with uncertain age ranges by specifying prior distributions (see **Supplementary Data 1**). We also specified a prior for the age of the root using a lognormal distribution with a mean of 0, a standard deviation of 3 and an offset of 83, which effectively imposes a minimum bound at year 1932 (the collection year of the oldest isolate in this study). Importantly, we conducted the analyses without sequence data to verify that the prior on the age of the root was not overly informative. Our analyses in BEAST were run with a Markov chain Monte Carlo length of 1×10^{10}, sampling every 5×10^{4} steps.

We used two molecular clock models; strict and relaxed uncorrelated lognormal (UCLD), and two demographic priors; the constant-size coalescent and the Bayesian coalescent Skyline, resulting in four model combinations. The analyses using the UCLD clock and the Skyline prior allowed for informal model testing. In particular, the constant-size coalescent is preferred over the Skyline if it is possible to trace a straight line through the reconstruction of the population size over time. Similarly, the coefficient of rate variation in the (UCLD) clock measures the degree of clock-like behaviour in the data, such that if its estimate is abutting zero, the strict clock is preferred. As a result, we selected the UCLD clock with constant population size as the preferred model.

To test for diversification in the population growth dynamics between different lineages, we investigated birth/death rates for nested subclades in the tree (the ratio of lineages that arise vs those that decline; a ratio >1 indicates population expansion, <1 indicates population decline. To do this, a multi-type birth-death model was fitted to a posterior sample of 100 phylogenetic trees inferred in BEAST under the Skyline tree prior, using the maximum likelihood implementation in TreePar v3.3 [11]. This model has eight parameters for a two-type process; birth rate within type 1 (λ_{11}), birth rate within type 2 (λ_{22}), birth rate from type 1 to type 2 (λ_{12}), birth rate from type 2 to type 1 (λ_{21}), death rate for type 1 (μ_1), death rate for type 2 (μ_2), sampling probability for type 1 (p_1), and sampling probability for type 2 (p_2). However, not all of these parameters are identifiable. For this reason we arbitrarily fixed the following parameters: $p_1 = p_2 = 0.01$, $\lambda_{11} = 15$, $\mu_2 = 4$. As such, the parameters for each lineage should be interpreted relative to those of the other lineage, and not in an absolute scale. We compared birth relative to death rates two lineages at a time (λ_{11}/μ_1 vs. λ_{22}/μ_2), which can be interpreted as the ratio between the number of lineages that arise to those that go extinct. We pruned the complete trees in two different ways. First we included only the horse-associated isolates and closely related human-associated CG23-I isolates. This revealed that the horse-associated lineage had a lower diversification rate. As such, we excluded the horse-associated lineage to compare diversification rates for the different human lineages (CG23-I and all other non CG23-I lineages).

The reliability of estimates of evolutionary rates and timescales is contingent on whether the data have temporal structure, which we verified using a date-randomisation test; i.e. we randomised the sampling times and repeated the analysis to generate rate estimates representing the expectation under no temporal structure [12]. The data are considered to have strong temporal structure, with reliable rate estimates, if the estimate with the correct

sampling times is not within the range of those obtained from the randomisations [13]. We conducted this test in both BEAST (20 randomisations) and LSD (100 randomisations).

Plasmid analyses

A novel circular plasmid sequence, pSB4816, was extracted from the genome assembly graph of equine isolate SB4816 using Bandage [14] and subsequently confirmed using hybrid assembly of the Illumina plus Nanopore reads as described above. The completed plasmid sequence was extracted and annotated using Prokka [15] followed by manual curation with reference to the Tn*21* reference sequence AF071413 [16]. The annotated sequence of pSB4816 has been deposited in Genbank (accession: MF363048. Conservation of this plasmid in other CG23 strains was assessed by mapping each read set to the concatenated pSB4816 contigs using RedDog, as described above for the virulence plasmid. Variation in the Tn*21*-like/AMR region was explored by manual inspection of assembly graphs.

To identify other likely AMR plasmids, we performed BLASTn searches within Bandage [14] to identify the location of AMR genes and plasmid replicons in the assembly graphs of all genomes in which acquired AMR genes were detected. In each case, the AMR genes were located in contigs that were linked in the assembly graph to contigs harbouring known plasmid rep genes (IncN or IncA/C2). However it was not possible to disentangle the complete plasmid sequences from the chromosome sequences in the assembly graphs, due to repeat copies of transposases and other sequences in these genomes. Fortunately plasmid MLST schemes are available for both IncN and IncA/C plasmids [17,18]; hence we used BLASTn searches to screen the assembly graphs for the plasmid MLST genes, and used this to (i) confirm the presence of the plasmid backbone in contigs linked to the AMR genes, and (ii) to genotype the plasmids via MLST.

Supplementary References

1. Wick, R. R., Judd, L. M., Gorrie, C. L. & Holt, K. E. Completing bacterial genome assemblies with multiplex MinION sequencing. *MGen* **3,** 1–7 (2017).
2. Wick, R. R., Judd, L. M., Gorrie, C. & Holt, K. E. Unicycler: Resolving bacterial genome assemblies from short and long sequencing reads. *PLoS Comput Biol* **13,** e1005595 (2017).
3. Loman, N., Quick, J. & Simpson, J. A complete bacterial genome assembled de novo using only nanopore sequencing data. *Nat. Methods* **12,** 733–735 (2015).
4. Garrison, E. & Marth, G. Haplotype-based variant detection from short-read sequencing. *arXiv* 1207.3907 (2012).
5. Langmead, B. & Saizberg, S. L. Fast gapped-read alignment with Bowtie 2. *Nat. Methods* **9,** 357–359 (2012).
6. Li, H. *et al.* The Sequence Alignment / Map format and SAMtools. *Bioinformatics* **25,** 2078–2079 (2009).
7. Zhou, Y., Liang, Y., Lynch, K. H., Dennis, J. J. & Wishart, D. S. PHAST: A Fast Phage Search Tool. *Nucleic Acids Res* **39,** W347–W352 (2011).
8. Rambaut, A., Lam, T. T., Carvalho, L. M. & Pybus, O. G. Exploring the temporal structure of heterochronous sequences using TempEst (formerly Path-O-Gen). *Virus Evol.* **2,** vew007 (2016).
9. To, T.-H., Jung, M., Lycett, S. & Gascuel, O. Fast Dating Using Least-Squares Criteria and Algorithms. *Syst Biol* **65,** 82–97 (2016).
10. Drummond, A. J., Suchard, M. A., Xie, D. & Rambaut, A. Bayesian Phylogenetics with BEAUti and the BEAST 1.7. *Mol Biol Evol* **29,** 1969–1973 (2012).
11. Stadler, T. Mammalian phylogeny reveals recent diversification rate shifts. *Proc Natl Acad Sci USA* **108,** 6187–6192 (2011).
12. Ramsden, C. *et al.* High Rates of Molecular Evolution in Hantaviruses. *Mol Biol Evol* **25,** 1488–1492 (2008).
13. Duchene, S., Duchene, D., Holmes, E. C. & Ho, S. Y. W. The Performance of the Date-Randomization Test in Phylogenetic Analyses of Time-Structured Virus Data. *Mol Biol Evol* **32,** 1895–1906 (2015).
14. Wick, R. R., Schultz, M. B., Zobel, J. & Holt, K. E. Genome analysis Bandage: interactive visualization of de novo genome assemblies. **31,** 3350–3352 (2015).
15. Seemann, T. Prokka: Rapid prokaryotic genome annotation. *Bioinformatics* **30,** 2068–2069 (2014).
16. Liebert, C. A., Hall, R. M. & Summers, A. O. Transposon Tn*21*, Flagship of the Floating Genome. *Microbiol Mol Biol Rev* **63,** 507–522 (1999).
17. Villa, L. *et al.* Multilocus sequence typing of IncN plasmids. *J Antimicrob Chemother* **66,** 1987–1991 (2011).
18. Hancock, S. J. *et al.* Identification of IncA/C Plasmid Replication and Maintenance Genes and Development of a Plasmid Multilocus Sequence Typing Scheme. *Antimicrob Agents Chemother* **61,** e01740-16 (2017).
