# Supplementary material for: Population genomics of hypervirulent Klebsiella pneumoniae clonal-group 23 reveals early emergence and rapid global dissemination
Source: Nat Commun. 2018 Jul 13;9:2703. doi: 10.1038/s41467-018-05114-7 (PMC6045662; doi:10.1038/s41467-018-05114-7)
Supplement: Supplementary file 3 — Description of Additional Supplementary Files [file 41467_2018_5114_MOESM3_ESM.pdf]

**Description of Additional Supplementary Files**

File Name: Supplementary Data 1
Description: Isolate information.

File Name: Supplementary Data 2
Description: Nucleotide mutations defining the globally distributed CG23-I sublineage.

File Name: Supplementary Data 3
Description: Nucleotide mutations defining the horse-associated sublineage.
